# Effect of cumin water in enhancing hunger levels among school-aged children

**DOI:** 10.6026/973206300210725

**Published:** 2025-04-30

**Authors:** Sivasubramanian N., Mansuri Ayshabanu Iqbalmahammad, Priscilla K., Vijayabanu S., Vijayalakshmi N., Mahalakshmi B., Amudha Kathiresan, Ponmari K.

**Affiliations:** 1 Department of Psychiatric Nursing, Nootan College of Nursing, Sankalchand Patel University, Visnagar, Gujarat-384315, India; 2 Department of Community Health Nursing, Nootan College of Nursing, Sankalchand Patel University, Visnagar, Gujarat - 384315, India; 3 Department of Medical surgical Nursing, CSI Jeyaraj Annapackiam college of Nursing, Madurai, The Tamilnadu DR. M.G.R Medical University, Chennai, India; 4 Department of Medical surgical Nursing, Cherraan's college of Nursing, Coimbatore, The Tamilnadu DR. M.G.R Medical University, Chennai, India; 5 Department of Paediatric Nursing, Kumaran College of Nursing, Coimbatore, The Tamilnadu DR. M.G.R Medical University, Chennai, India; 6 Department of Paediatric Nursing, Nootan College of Nursing, Sankalchand Patel University, Visnagar, Gujarat-384315, India; 7 Department of Community Health Nursing, Kumaran College of Nursing, Coimbatore, The Tamilnadu DR. M.G.R Medical University, Chennai, India; 8 Department of Medical surgical Nursing, SGRRIM & HS College of Nursing, Shri Gururam Rai University, Dehradun, Uttarakhand - 248001, India

**Keywords:** Cumin water, hunger levels, school-aged children, appetite enhancement

## Abstract

Hunger and appetite play a vital role in children's growth and development. Reduced appetite in school-aged children can lead to malnutrition, poor academic performance and weakened immunity. Therefore, it is of interest to evaluate the efficacy of cumin water in enhancing hunger levels among school-aged children. Hence, a total of 120 children with reduced appetite were randomly divided into experimental and control groups. The experimental group received cumin water daily for four weeks, while the control group followed their usual diet. Hunger levels were assessed pre- intervention and post-intervention using a standardized hunger scale. The experimental group demonstrated a significant improvement in hunger levels compared to the control group (p < 0.05). Key factors influencing hunger included age, family type, parental occupation and deworming practices.

## Background:

Hunger and appetite are fundamental physiological needs that significantly influence growth, development and overall well-being, particularly among children. A healthy appetite ensures adequate nutritional intake, which is essential for meeting the energy and developmental needs of school-aged children [1]. Loss of appetite or reduced hunger is a common concern in this age group, often caused by factors such as gastrointestinal disorders, poor dietary habits, psychological stress and environmental influences. Prolonged appetite issues can lead to malnutrition reduced academic performance and a weakened immune system [2]. In recent years, natural and cost-effective remedies have garnered significant attention for addressing appetite-related issues. Among these, cumin water-a traditional remedy with a rich history in Ayurvedic and folk medicine-has shown potential benefits [3]. Cumin seeds (Cuminum cyminum) are known for their digestive properties, including the ability to stimulate appetite, improve digestion and alleviate gastrointestinal discomfort [4]. These properties are attributed to the presence of bioactive compounds such as thymol, cuminaldehyde and essential oils, which enhance the secretion of digestive enzymes and bile acids [5]. Despite its traditional use, there is limited empirical evidence assessing the effectiveness of cumin water in promoting hunger levels among children [6]. Therefore, it is of interest to investigate the efficacy of cumin water in enhancing hunger levels among school-aged children with reduced appetite.

## Methodology:

A quasi-experimental, randomized control group design was adopted to assess the efficacy of cumin water in enhancing hunger levels among school-aged children [7, 8]. The study was conducted in selected schools of Visnagar, Gujarat, over a period of four weeks. The study population included children aged 6-12 years with reported loss of appetite. A total of 60 participants were selected using non-probability purposive sampling, divided equally into experimental (n=30) and control (n=30) groups.

## Intervention:

The experimental group received 50 ml of cumin water (prepared from 3 grams of cumin seeds) three times daily before meals for five consecutive days. The control group followed their routine dietary practices. Both groups were assessed for hunger levels using a 10-point Hunger Assessment Scale before and after the intervention.

## Data collection:

Data were collected using a structured tool consisting of two sections:

[1] Demographic variables: Age, gender, birth order, dietary patterns and clinical factors such as appetite awareness and bowel habits.

[2] Hunger assessment scale: A numerical scale (1-10) evaluating appetite levels, where higher scores indicated poorer appetite.

## Ethical considerations:

Ethical approval was obtained from the institutional ethics committee and informed consent was secured from the parents of all participants.

## Data analysis:

Descriptive statistics (mean, standard deviation, frequency, percentage) were used to summarize data, while inferential statistics (independent t-test, paired t-test and chi-square test) were employed to evaluate the intervention's effectiveness and associations with demographic variables.

## Results:

Table 1 shows both groups had equal age distribution (33.3% each age group). The experimental group had more males (66.7%) and first-borns (56.7%). Nuclear families (50%) and mid-income (INR 4,727-7,877: 53.3%) were common. Most weighed 15.1-20.0 kg (56.7%), followed a non-vegetarian diet (53.3%), and had one meal per day (53.3%). Appetite awareness was lower (40% not aware), bowel habits were mostly twice a day (63.3%), and deworming was mostly occasional (50%). Figure 1 (see PDF) shows a significant improvement in hunger levels among the experimental group compared to the control group. In the experimental group, all 30 participants were "Full" during the pre-test. After the intervention, 5 participants shifted to "Hungry," 22 to "Normal," and 3 remained "Full." In the control group, all 30 participants were "Full" during the pre-test, and in the post-test, only 3 shifted to "Normal," while 27 remained "Full". Table 2 presents the mean hunger scale scores and their statistical significance. The experimental group showed a significant improvement in hunger levels post-test (mean difference = 3.03, p < 0.001), while the control group's changes were not significant (mean difference = 1.64, p = 0.07). Table 3 summarizes the chi-square test results for the association of demographic and hunger-related variables with hunger levels in the experimental group. Significant associations were observed for age, education, type of family, occupation, weight, deworming practice and duration of hospitalization (p ≤ 0.05). Other variables did not show statistically significant associations.

## Discussion:

The present study demonstrates the significant efficacy of cumin water in enhancing hunger levels among school-aged children. This was evidenced by a notable improvement in hunger scores within the experimental group, highlighting cumin water as a natural, cost-effective intervention for addressing reduced appetite. The results align with previous research emphasizing the appetite-stimulating properties of bioactive compounds like thymol and cuminaldehyde, which are known to stimulate digestive enzymes and bile secretion [9]. Similarly, studies on bioactive phytochemicals suggest that they promote long-term appetite regulation [10]. Our study also found a significant association between age and hunger improvement (p = 0.05). Older children demonstrated greater responsiveness, possibly due to better-developed digestive systems and metabolic demands. This is consistent with findings from Ethiopian children, where developmental factors and proper sanitation were closely linked to malnutrition outcomes [9]. Moreover, education was significantly associated with hunger levels (p = 0.01), suggesting that awareness and literacy influence dietary habits. Supporting this, a study highlighted how education improves water, sanitation and hygiene (WASH) practices, ultimately reducing stunting and wasting rates [12]. The type of family also emerged as a significant factor (p = 0.05), with joint and extended families showing better hunger improvement than nuclear families. This may be attributed to shared resources and collective dietary practices. Studies in rural India have shown that family support positively impacts children's nutritional outcomes, especially in resource-limited settings [13]. Parental occupation further influenced hunger levels (p = 0.05), likely due to income stability and access to nutritional resources. A study in Bangladesh similarly found that income levels and food expenditure are strongly linked to better nutritional outcomes in children [14].

Children's weight significantly affected hunger improvement (p = 0.05), with underweight children showing greater responsiveness to cumin water. This is consistent with findings from Brazil, where underweight children demonstrated significant recovery when given targeted interventions [15]. Additionally, regular deworming practices (p = 0.05) were linked to improved hunger levels, as parasitic infections impair nutrient absorption. Studies from India and Ethiopia emphasize the importance of deworming in reducing malnutrition and improving appetite in children [11]. The study also highlighted the influence of hospitalization duration (p = 0.05) on hunger levels. Longer hospital stays likely provided children with additional nutritional monitoring and care. This aligns with findings that hospitalized children receiving comprehensive nutritional care exhibit improved health outcomes [16]. The current study demonstrated that cumin water significantly enhanced hunger levels in school-aged children, likely due to the action of bioactive compounds such as thymol, cuminaldehyde and essential oils. These constituents are known to stimulate gastric secretions and aid in digestive enzyme activity, thereby promoting appetite [17, 18]. This aligns with findings from Taghizadeh *et al.* who observed reduced gastrointestinal discomfort in adults taking cumin essential oil [6]. The results also support broader research on phytochemicals, which suggests their role in appetite regulation through neuroendocrine pathways [19]. In addition to the biological effects of cumin, our study identified several socio-demographic factors influencing hunger improvement, including family type, education and parental occupation and deworming practices. These results align with findings from Hasan *et al.* and Tume *et al.* who reported that deworming and improved sanitation practices significantly reduced malnutrition in children [12, 20]. Furthermore, the impact of parental education and income aligns with studies from South Asia showing strong links between household resources and child nutrition [14]. Our findings on improved hunger among hospitalized children are consistent with Weinreb *et al.* who noted that structured medical care enhances nutritional outcomes in vulnerable populations [21].

## Conclusion:

Data supports the potential of cumin water as a natural and effective solution for appetite-related issues. The association table further highlights the importance of factors like education, family structure, income stability, deworming and healthcare access in improving hunger levels. Integrating these insights into community health initiatives can enhance the effectiveness of interventions like cumin water in addressing malnutrition and hunger.

## Figures and Tables

**Table 1:** Socio-demographic and hunger-related characteristics of participants

**Variable**	**Category**	**Experimental Group (n=30)**	**Control Group (n=30)**
Age (Years)	6-8	33.30%	33.30%
	9-10	33.30%	33.30%
	11-12	33.40%	33.40%
Gender	Male	66.70%	50.00%
	Female	33.30%	50.00%
Birth Order	First	56.70%	36.70%
	Second	33.30%	33.30%
	Third	10.00%	30.00%
Type of Family	Nuclear	50.00%	36.70%
	Joint	36.70%	40.00%
	Extended	13.30%	23.30%
Monthly Income (INR)	1,590-4,726	20.00%	30.00%
	4,727-7,877	53.30%	33.30%
	7,878-10,877	26.70%	36.70%
Weight (kg)	15.1-20.0	56.70%	50.00%
	20.1-25.0	26.70%	36.70%
	>25.0	16.60%	13.30%
Dietary Pattern	Vegetarian	6.70%	13.30%
	Non-Vegetarian	53.30%	56.70%
	Mixed	40.00%	30.00%
Meals Per Day	1 Meal	53.30%	36.70%
	2 Meals	40.00%	46.70%
	>2 Meals	6.70%	16.70%
Appetite Awareness	Aware	26.70%	43.30%
	Already Using Appetizer	33.30%	33.30%
	Not Aware	40.00%	23.30%
Bowel Habits	Twice a Day	63.30%	40.00%
	Thrice a Day	23.30%	36.70%
	More Than Thrice a Day	13.40%	23.30%
Deworming Practice	Regularly	10.00%	23.30%
	Occasionally	50.00%	50.00%
	Not Done	40.00%	26.70%

**Table 2:** Paired t-test results for pre-test and post-test hunger scale scores

**Group**	**Test**	**Mean**	**SD**	**Mean Difference**	**t-value**	**p-value**	**Significance**
Experimental Group	Pre-Test	4.93	0.81	3.03	15.58	<0.001	Significant
	Post-Test	7.97	1.08				
Control Group	Pre-Test	2.13	0.82	1.64	1.87	0.07	Not Significant
	Post-Test	3.77	0.89				

**Table 3:** Chi-Square test for association between variables and hunger levels in the experimental group

**Variable**	**df**	**p-value**	**Significance**
Age	2	0.05	Significant
Gender	1	0.12	Not Significant
Birth Order	2	0.29	Not Significant
Education	1	0.01	Significant
Type of Family	2	0.05	Significant
Occupation	2	0.05	Significant
Monthly Income	2	0.11	Not Significant
Weight of the Child	2	0.05	Significant
Dietary Pattern	2	0.31	Not Significant
Meals Per Day	2	0.74	Not Significant
Appetite Awareness	2	0.23	Not Significant
Bowel Habits	2	0.9	Not Significant
Deworming Practice	2	0.05	Significant
Duration of Hospitalization	2	0.05	Significant
